# Clopidogrel versus Ticagrelor for Secondary Prevention after Coronary Artery Bypass Grafting

**DOI:** 10.3390/jcm8010104

**Published:** 2019-01-17

**Authors:** Hyoung Woo Chang, Hee Jung Kim, Jae Suk Yoo, Dong Jin Kim, Kwang Ree Cho

**Affiliations:** 1 Department of Thoracic and Cardiovascular Surgery, Seoul National University Bundang Hospital, Gyeonggi-do 13620, Korea; changhw@snubh.org; 2 Department of Thoracic and Cardiovascular Surgery, Sejong General Hospital, Gyeonggi-do 14754, Korea; heejung440@hanmail.net (H.J.K.); mdyoo77@gmail.com (J.S.Y.); shallbe@hanmail.net (D.J.K.)

**Keywords:** acute coronary syndrome (ACS), secondary prevention, coronary artery bypass grafting, clopidogrel, antiplatelet therapy

## Abstract

We sought to evaluate the outcomes of postoperative three-month dual antiplatelet therapy in patients with non-ST-elevation acute coronary syndrome (NSTE-ACS) following off-pump coronary artery bypass grafting (OPCAB) with exclusively arterial grafts. Between 2013–2016, dual antiplatelet therapy (DAPT) with either aspirin + clopidogrel (ASA + CPD group, *n* = 100) or aspirin + ticagrelor (ASA + TCG group, *n* = 169) was prescribed postoperatively in 269 NSTE-ACS patients after total arterial OPCAB. Patients with indications for other oral anticoagulants were excluded from the study. Three-month DAPT was completed in 259 patients (96%); ASA + CPD group (*n* = 94) vs. ASA + TCG group (*n* = 165). A one-to-one propensity score matching was performed. Unadjusted comparison between the groups showed no significant difference in overall survival (*P* = 0.253) and composite outcome of major adverse cerebrovascular and cardiovascular event (MACCE) and major bleeding (*P* = 0.276). The rate of freedom from composite outcome at one year in the ASA + CPD and ASA + TCG groups was 91 ± 3% and 93 ± 2%, respectively. In multivariable analysis, being in the ASA + TCG group did not increase the risk of the composite outcome of MACCE and major bleeding (*P* = 0.972, hazard ratio: 1.0, 95% confidence interval: 0.4–2.3). Propensity score-matched comparison (76 pairs) showed no significant difference in the overall survival (*P* = 0.423) and composite outcome between the groups (*P* = 0.442). In the setting of exclusive arterial grafting, post-OPCAB three-month DAPT showed acceptable outcomes in patients with NSTE-ACS. There was no significant difference in overall survival or composite outcome of MACCE and major bleeding between the ASA + CPD and ASA + TCG groups.

## 1. Introduction

According to the latest guidelines for the treatment of coronary artery disease, dual antiplatelet therapy (DAPT) using aspirin (acetylsalicylic acid, ASA) with either clopidogrel (CPD) or ticagrelor (TCG) is recommended to maintain the patency of the stent following percutaneous coronary intervention (PCI) [1]. Especially in non-ST-elevation acute coronary syndrome (NSTE-ACS), it is recommended that DAPT be performed with a combination of ASA + TCG rather than ASA + CPD [1,2,3]. On the other hand, DAPT for 1 year is also recommended for secondary prevention after off-pump coronary artery bypass grafting (CABG) [4]. However, the choice between ASA + CPD and ASA + TCG remains unclear for CABG. 

Current recommendations for DAPT after CABG are based on a heterogeneous collection of CABG strategies. The biggest two axes for distinguishing the CABG strategies are on-pump vs. off-pump, and exclusively arterial vs. artery and vein mixed graft strategies. The patient is not fully heparinized during off-pump CABG, and therefore more intensive secondary prevention has been suggested [4]. On the other hand, despite the documented superiority of total arterial revascularization in terms of long-term patency, the majority of surgeons still use vein grafts [5,6,7]. Additionally, the currently available studies on DAPT after CABG mostly have used mixed arterial and venous grafts. However, in our institute, the protocol includes off-pump CABG using bilateral internal thoracic arteries (ITAs). The aim of this study was to compare the outcomes of postoperative three-month DAPT after total arterial off-pump CABG in patients with NSTE-ACS.

## 2. Material and Methods

### 2.1. Study Design

**Figure 1:**
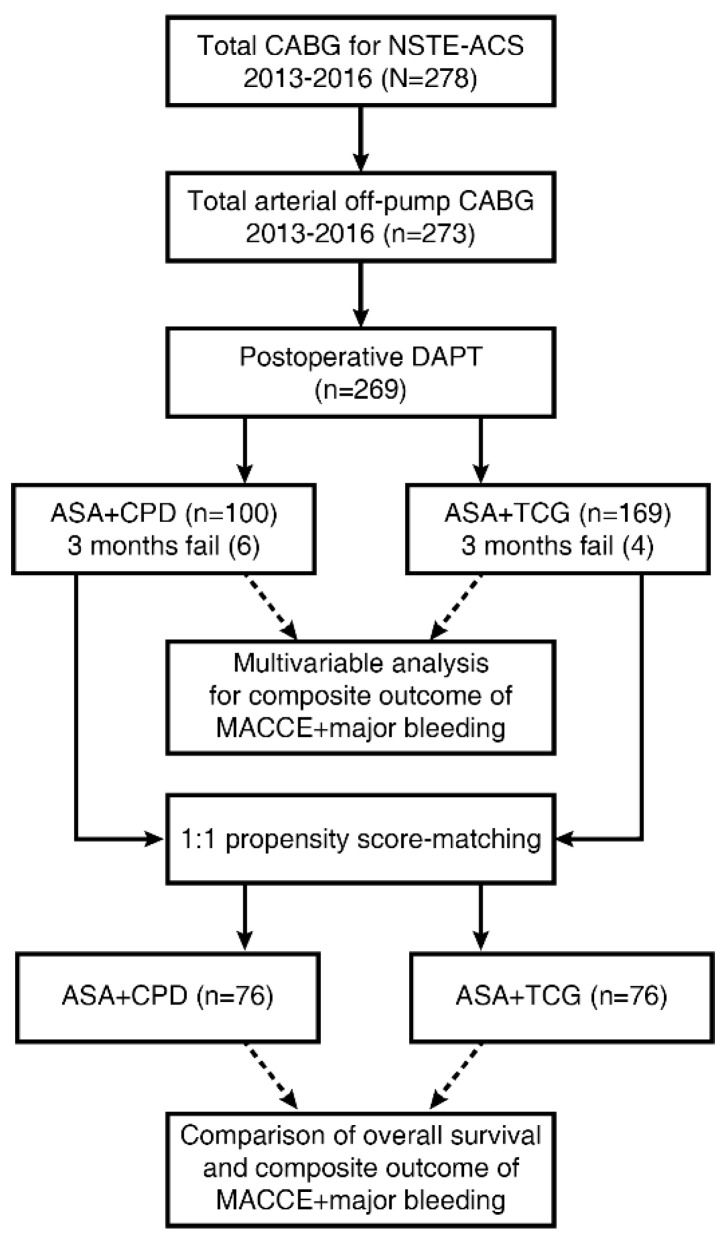
Flow diagram of the study design and analyses. ASA, acetylsalicylic acid; CABG, coronary artery bypass grafting; CPD, clopidogrel; DAPT, dual antiplatelet therapy; MACCE, major adverse cerebrovascular and cardiovascular event; TCG, ticagrelor.

This is a retrospective observational study, and data were collected through review of electronic medical records. The institutional review board of the hospital approved this study, and informed consent from patients was waived based on the retrospective nature of this study (IRB No.:2017-802). From January 2013 to December 2016, a total of 278 off-pump CABGs were performed in patients with NSTE-ACS in our hospital. Nine patients were excluded from the study because saphenous veins were used in five patients, and DAPT was not initiated in four patients. DAPT was not initiated in the four patients because of the use of rivaroxaban due to atrial fibrillation (*n* = 1), hematochezia (*n* = 1), mediastinitis (*n* = 1), and end-stage renal disease with low hemoglobin (*n* = 1). In this study, data from 269 patients (98%) who underwent total arterial revascularization and underwent DAPT for at least 1 day were reviewed (Figure 1). Patients with indications for other oral anticoagulants were excluded from the study.

### 2.2. Surgical Techniques

In this study, all patients underwent off-pump CABG. Following full median sternotomy, bilateral ITAs were harvested with skeletonization from the level of the 1st rib to 1–2 cm from the bifurcation of superior epigastric and musculophrenic arteries after the latter was ligated and divided. Branches of the ITAs were divided after metal clipping. Commonly, the right ITA was used as the free graft and the left ITA was used as the in situ graft. Before completely separating the ITA from the chest wall, an initial dose of heparin (1.5 mg/kg) was administered. Activated clotting time (ACT) was maintained above 300 s until the end of the last anastomosis. After opening the pericardium, the right ITA was anastomosed (end-to-side) with the left ITA in situ at the level of the pulmonary conus to construct a Y-composite graft. The distal end of the in situ left ITA was anastomosed with the left anterior descending artery in most of the cases, and coronary arteries on the lateral and inferior walls were revascularized with the right ITA sequentially. Graft patency was evaluated using transit-time flowmeter for each anastomosis. After finishing the last anastomosis, protamine was administered to neutralize the heparin.

### 2.3. Perioperative Management

Aspirin was used until the morning of surgery and resumed within 6 hours after the surgery. Heparin was discontinued 6 hours before the surgery in patients who required a preoperative heparin infusion. Even in patients who were loaded with antiplatelet agents to try percutaneous coronary intervention, we did not postpone the surgeries. In patients with recent antiplatelet agent loading, platelet concentrate was prepared before the surgery and transfusion was initiated after the intraoperative bolus of heparin injection. After surgery, ACT was checked again, and a small amount of protamine (10–20 mg) was administered as needed. However, no antifibrinolytic agent (e.g., tranexamic acid) was used. Heparin continuous infusion was resumed once the active bleeding ceased.

### 2.4. Postoperative Dual Antiplatelet Therapy

Our institute has been following a protocol of 3-month DAPT after off-pump CABG for NSTE-ACS since 2012. DAPT was initiated within 24 h of CABG. In patients who were not extubated on postoperative day 1, antiplatelet agents were administered via Levin tubes. For the DAPT regimen, a combination of ASA + CPD or ASA + TCG was used; aspirin 100 mg daily + clopidogrel 75 mg daily, or aspirin 100 mg daily + ticagrelor 90 mg twice a day. ASA + CPD was previously used; however, since March 2015, ASA + TCG was also used due to the change in the guidelines for the treatment of NSTE-ACS according to the results of the Platelet Inhibition and Patient Outcomes (PLATO) Trial. In the absence of a specific contraindication to ticagrelor, ASA + TCG was used for 3 months after CABG. Without any specific reason, DAPT was discontinued after a 3-month period and aspirin alone was used indefinitely. If there was a need to maintain DAPT afterwards, we changed to ASA + CPD combination regardless of the initial DAPT regimen.

### 2.5. Follow-Up

The mean follow-up period was 1.8 years (max; 4.61 years). In the outpatient department, patients were asked about symptoms such as chest pain and dyspnea. When the physician deemed necessary, the patient was admitted, and further studies were performed. Coronary CT angiography was routinely performed at one year after the surgery to evaluate graft patency, unless a patient had renal dysfunction (serum creatinine ≥ 1.3 mg/dL).

In this study, the definition of a major adverse cardiovascular and cerebrovascular event (MACCE) included death from any cause, new myocardial infarction, target vessel revascularization (TVR), or stroke. ‘Major bleeding’ was defined as fatal bleeding, intracranial or retroperitoneal bleeding, and any bleeding resulting in hemoglobin decrement of >2 g/dL.

### 2.6. Statistical Analyses

A summary diagram of the process of analyses is presented in Figure 1. PASW 20 (IBM Inc., Armonk, NY, USA) and R (version 3.1.1, R Foundation for Statistical Computing) were used. Student *t*- and chi-square tests were used for comparison of continuous and categorical variables, respectively. A Cox proportional hazard ratio model was used to identify independent predictors of the composite endpoint (MACCE + major bleeding).

To overcome the differences in the baseline characteristics between the two groups, propensity score matching was used. A logistic regression model was constructed to calculate the propensity score for each case on the disposition in either of the two groups. Variables in this propensity score model included age, sex, body surface area, diabetes, hypertension, creatinine clearance, atrial fibrillation, old myocardial infarction, recent percutaneous coronary intervention, current smoking, EuroSCORE II, non-ST-elevation myocardial infarction, left main artery disease, number of anastomoses, and emergency surgery. Using the propensity scores derived from this model, one-to-one propensity score matching was performed with the use of the nearest neighbor method with a caliper of 0.1. In order to compare survival data between a matched population, Kaplan-Meier survival curves were drawn, and a Cox proportional hazard ratio model with matching was used (package ‘coxph’ with ‘strata’).

## 3. Results

### 3.1. Patient Characteristics and Operative Data

The mean age of the patients was 64 ± 10 years, and 201 patients (75%) were male. Baseline patient characteristics are presented in Table 1. The operative data are summarized in Table 2. There were significantly larger numbers of patients with NSTEMI in the ASA + TCG group (*P* = 0.003). Operative data are shown in Table 2 and none were significantly different between the groups.

### 3.2. Postoperative Dual Antiplatelet Therapy

**Figure 2:**
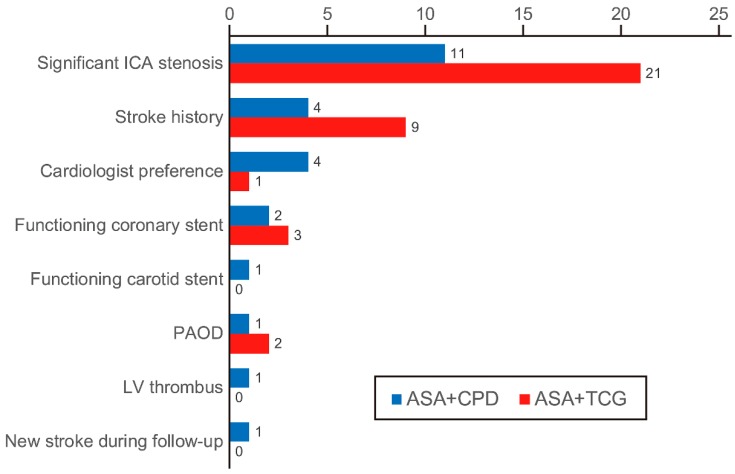
The reasons of continuing dual antiplatelet therapy (DAPT) after 3 months. Cardiologist preference: when the patients were followed-up by cardiologists and the latter decided to maintain DAPT after predetermined 3-month period. ASA, acetylsalicylic acid; CPD, clopidogrel; ICA, internal carotid artery; LV, left ventricle; PAOD, peripheral artery occlusive disease; TCG, ticagrelor.

Of the 269 patients, 259 patients (96%) completed postoperative DAPT for three months (Figure 1), of which 94 patients (94%) were in the ASA + CPD group vs. 165 patients (98%) in the ASA + TCG group. The reasons for not completing the 3-month DAPT were early death (*n* = 3), shifting to warfarin (*n* = 2), reduction in hemoglobin (*n* = 2), and gastrointestinal bleeding (*n* = 2). As shown in Figure 2, of the 259 patients who completed the three-month DAPT, 61 patients continued it after three months for various reasons. The proportion of patients who continued DAPT after three months did not differ significantly between the groups; 25/94 patients (27%) in the ASA + CPD group vs. 36/165 patients (22%) in the ASA + TCG group (*P* = 0.447). The patients in the ASA + TCG group who continued DAPT after three months were shifted to ASA + CPD combination therapy because of the inconvenient regimen that TCG should be taken twice a day.

### 3.3. Overall Outcomes and Multivariable Analysis

**Figure 3:**
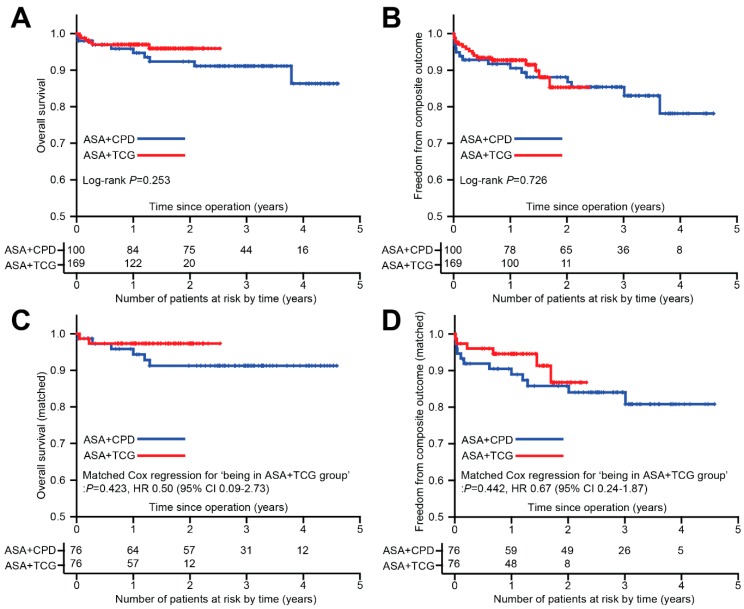
Survival analysis (Kaplan–Meier analyses) for all 269 patients and matched patients. (**A**) overall survival (all patients). (**B**) composite endpoint of major adverse cerebrovascular and cardiovascular event (MACCE) + major bleeding (all patients). (**C**) overall survival (propensity score-matched). (**D**) composite endpoint of MACCE and major bleeding (propensity score-matched). Crosshair means censored data. ASA, acetylsalicylic acid; CI, confidence interval; CPD, clopidogrel; OR, odds ratio; TCG, ticagrelor.

There was a total of 32 composite outcomes of MACCE or major bleeding; 16 in each group. A detailed description of the composite outcomes is provided in Table 3. The most frequent composite outcome was gastrointestinal bleeding (*n* = 12). Other outcomes included death from any cause (*n* = 11), stroke (*n* = 5), cerebral or cerebellar hemorrhage (*n* = 2), new myocardial infarction (*n* = 1), and target vessel revascularization (*n* = 1). No significant intergroup difference was found. Unadjusted comparison for overall survival and composite outcomes is shown in Figure 3A,B. Log-rank tests showed no significant difference in the overall survival or composite outcomes. Coronary CT angiography at one year postoperatively was performed in 128 patients (48%); 55/100 in the ASA + CPD group and 73/169 in the ASA + TCG group. Of a total of 477 anastomoses, 466 (98%) were patent; 196/199 in the ASA + CPD group vs. 270/278 in the ASA + TCG group (*P* = 0.374).

The variables which had a P value less than 0.2 in univariate analyses were included in multivariable analyses for composite outcomes with the variable ‘use of ticagrelor’ (Table 4). As a result, only ‘hemodialysis’ was an independent risk factor (*P* = 0.046, hazard ratio (HR): 3.96, 95% confidence interval (CI): 1.02–15.33) of the composite outcome of MACCE and major bleeding. ‘Use of ticagrelor’ was not an independent predictor of the composite outcome (*P* = 0.972, HR: 0.99, 95% CI: 0.43–2.28).

### 3.4. Propensity Score Matched Analyses

After one-to-one propensity score matching, 76 pairs were identified. Baseline characteristics that had large imbalance (standardized mean difference >25%) before matching showed acceptable balance after matching (Table 5). Both overall survival and the composite outcome of MACCE or major bleeding showed no significant difference between the groups (Figure 3C,D) with matched Cox regression analysis.

## 4. Discussion

Clopidogrel, prasugrel, and ticagrelor are some of the P2Y12 inhibiting antiplatelet agents used in combination with aspirin. Clopidogrel has the longest history; it was found to have a number of advantages in combination with aspirin compared with aspirin alone in post-PCI patients. Ticagrelor has recently emerged as one of the commercially available P2Y12 inhibitors. It has rapid onset and higher potency than clopidogrel. Unlike clopidogrel, ticagrelor rarely results in resistance to the drug. Ticagrelor needs to be taken twice a day, and a substantial number of patients complain of dyspnea. However, it has already been established in the guidelines that it is better to prescribe DAPT in the form of combined ASA + TCG than ASA + CPD after invasive treatment (PCI or surgery) in patients with NSTE-ACS [1]. Following CABG, many studies have shown that postoperative DAPT with ASA + CPD is better than ASA alone in terms of clinical endpoints such as MACCE. The necessity of DAPT after CABG in patients with NSTE-ACS is documented in the guidelines [2,4].

There has been reports that evaluated the efficacy of DAPT after CABG. However, our study is different from previous reports in several aspects. First, the patient population in the previous studies underwent CABG using venous grafts [8,9,10,11,12]. Many high-volume CABG centers (>100 CABGs annually) still use saphenous veins as their main grafts [6,7]. The subjects in our study are unique because they received total arterial CABG using bilateral ITAs. Second, our cases consist of only off-pump CABGs. Off-pump and on-pump CABG need to be treated differently in terms of DAPT. One of the recent guidelines made recommendations specific to off-pump CABG because off-pump CABG is more likely to induce a hypercoagulable state after surgery than on-pump CABG [4]. Off-pump CABG is less likely to induce platelet dysfunction or coagulopathy that can be brought about by extracorporeal circulation [13,14]. There is a paucity of papers about DAPT dealing with only off-pump CABG without manipulating the ascending aorta [15].

In this unique setting, we compared the results of two DAPT regimes in 269 patients with NSTE-ACS. Two DAPT regimens were applied for three months postoperatively. Propensity score matched analyses were performed, and no statistically significant difference in the clinical outcomes was found between ASA + CPD and ASA + TCG combination therapy.

There are some speculations as to why these results were obtained. Firstly, the results may not have been affected by the type of P2Y12 inhibitor because we used only arterial grafts rather than venous grafts. Secondly, it may be because our study included an exclusively Korean population. The sub-analysis of PLATO trial subjects showed that the superiority of ASA + TCG over ASA + CPD is unclear in Asian populations [16].

There are limitations to this study. This study was a retrospective observational study with a relatively small sample size. Due to the retrospective nature, this study is hypothesis-generating rather than conclusion forming. Additionally, since the ASA + TCG combination was started later, there is a difference in the follow-up period between the two groups. However, the number of patients in the ASA + TCG group were more than 1.5 times the number of patients in the ASA + CPD group. Another thing to be commented on is that the duration of postoperative DAPT was three months, which we have followed before the introduction of one-year DAPT after off-pump CABG. Finally, although this study evaluated important clinical endpoints such as MACCE and major bleeding, we drew insufficient conclusions about graft patency because approximately half of the patients did not undergo coronary CT angiography at one year.

## 5. Conclusions

In conclusion, following total-arterial off-pump CABG in patients with NSTE-ACS, postoperative DAPT using the combination of ASA + CPD vs. ASA + TCG did not result in a significant difference in the overall survival and composite outcomes of MACCE and major bleeding. Prospective randomized studies are needed to identify optimal duration and regimen of DAPT following total-arterial CABG.

## Figures and Tables

**Table 1:** Baseline characteristics of the patients.

Variables	ASA + CPD (*n* = 100)	ASA + TCG (*n* = 169)	*P* Value
Age, years	64 ± 10	64 ± 10	0.865
Male sex, *n* (%)	80 (80%)	121 (72%)	0.147
Diabetes, (%)	52 (52%)	88 (51%)	0.900
HbA1c, (%)	6.6 ± 1.3	6.5 ± 1.3	0.540
Hypertension, *n* (%)	66 (66%)	89 (53%)	0.041 ^a^
Dyslipidaemia, *n* (%)	100 (100.0%)	168 (99%)	>0.999
CVA (<1 year), *n* (%)	1 (1%)	7 (4%)	0.265
Old MI (>1 year), *n* (%)	13 (13%)	13 (8%)	0.200
Serum creatinine (mg/dL)	1.9 ± 2.7	1.0 ± 0.5	0.001 ^a^
Hemodialysis, *n* (%)	10 (10%)	1 (1%)	<0.001 ^a^
Poor mobility, *n* (%)	2 (2%)	2 (1%)	0.632
Recent PCI (<3 months), *n* (%)	5 (5%)	13 (8%)	0.458
Abnormal brain CT, *n* (%)	17/58(29%)	34/115 (30%)	>0.999
Abnormal carotid CT, *n* (%)	25/83 (30%)	47/122 (39%)	0.236
PAOD, *n* (%)	6 (6%)	4 (2%)	0.181
COPD, *n* (%)	7 (7%)	0 (0%)	0.001 ^a^
Current smoker, *n* (%)	31 (31%)	47 (28%)	0.581
Atrial fibrillation, *n* (%)	11 (11%)	7 (4%)	0.042 ^a^
EuroSCORE II, (%)	10 ± 9	8 ± 7	0.093
LV ejection fraction, (%)	52 ± 14	50 ± 14	0.136
LV dysfunction, (<35%)	13 (13%)	25 (15%)	0.856
PASP (mmHg)	27 ± 6	27 ± 8	0.787
Unstable angina, *n* (%)	51 (51%)	55 (32%)	0.002 ^a^
NSTEMI, *n* (%)	49 (49%)	114 (68%)	0.002 ^a^
Left main disease, *n* (%)	33 (33%)	46 (27%)	0.334
1-vessel disease, *n* (%)	0	8 (5%)	0.028 ^a^
2-vessel disease, *n* (%)	25 (25%)	33 (20%)	0.357
3-vessel disease, *n* (%)	72 (72%)	129 (76%)	0.469

ASA, acetylsalicylic acid; COPD, chronic obstructive pulmonary disease; CPD, clopidogrel; CT, computed tomography; CVA, cerebrovascular accident; LV, left ventricle; MI, myocardial infarction; NSTEMI, non-ST-elevation myocardial infarction; PAOD, peripheral artery occlusive disease; PASP, pulmonary artery systolic pressure; PCI, percutaneous coronary intervention; TCG, ticagrelor. ^a^ *P* value less than 0.05.

**Table 2:** Operative data.

Variables	ASA + CPD (*n* = 100)	ASA + TCG (*n* = 169)	*P* Value
Urgent operation, *n* (%)	4 (4%)	6 (4%)	>0.999
Number of anastomoses	3.6 ± 0.9	3.7 ± 1.0	0.206
Only bilateral ITA for grafting, *n* (%)	100 (100%)	168 (99%)	>0.999
Perioperative IABP support, *n* (%)	14 (14%)	14 (8%)	0.152
Postoperative ECMO support, *n* (%)	1 (1%)	0	0.372
Re-exploration for bleeding, *n* (%)	5 (5%)	4 (2%)	0.299
Early mortality (<30 days), *n* (%)	2 (2%)	2 (1%)	0.630

ASA, acetylsalicylic acid; CPD, clopidogrel; ECMO, extracorporeal membrane oxygenation; IABP, intra-aortic balloon pump; ITA, internal thoracic artery; TCG, ticagrelor.

**Table 3:** List of patients with major adverse cerebrovascular and cardiovascular event (MACCE) or major bleeding.

ASA + CPD (16 Outcomes in 100 Patients)			ASA + TCG (16 Outcomes in 169 Patients)		
Sex/Age	Time since Surgery (Years)	Explanation	Sex/Age	Time since Surgery (Years)	Explanation
M/61	0.00	Stroke	M/50	0.01	Stroke
M/60	0.01	Stroke	F/75	0.01	Stroke
M/61	0.02	Death	F/75	0.03	Upper GI bleeding
M/52	0.02	Cerebral hemorrhage	M/56	0.03	Upper GI bleeding
M/74	0.03	Death	F/78	0.08	Upper GI bleeding
F/62	0.04	Upper GI bleeding	F/77	0.16	Death
F/74	0.11	Upper GI bleeding	M/70	0.22	Death
M/67	0.16	Upper GI bleeding	M/64	0.28	Death
M/67	0.61	Death	M/80	0.33	Upper GI bleeding
M/62	1.00	Death	M/48	0.36	Upper GI bleeding
M/86	1.20	Death	F/75	0.41	New MI during follow-up
M/73	1.29	Death	M/67	0.68	Stroke
M/68	2.01	Upper GI bleeding	M/79	1.28	Death
M/79	2.08	Death	F/72	1.45	Lower GI bleeding
M/61	3.01	Cerebellar hemorrhage	M/61	1.51	TVR (redo CABG)
M/58	3.64	Upper GI bleeding	M/60	1.69	Upper GI bleeding

ASA, acetylsalicylic acid; CABG, coronary artery bypass grafting; CPD, clopidogrel; F, female; GI, gastrointestinal; M, male; MI, myocardial infarction; TCG, ticagrelor; TVR, target vessel revascularization.

**Table 4:** Multivariable analysis (Cox proportional hazard ratio model) for composite outcomes of major adverse cerebrovascular and cardiovascular event (MACCE) and major bleeding.

Variables	Univariate Analysis *P* Value	Multivariable Analysis *P* Value	HR (95% CI)
Age	0.114	0.566	1.01 (0.97–1.06)
Sex	0.613		
Use of ticagrelor	0.727	0.972	0.99 (0.43–2.28)
Body surface area	0.044	0.406	0.35 (0.03–4.19)
Diabetes	0.157	0.473	1.32 (0.62–2.85)
Hypertension	0.245		
Hemodialysis	0.032	0.046 ^a^	3.96 (1.02–15.33)
Atrial fibrillation	0.076	0.183	1.97 (0.73–5.33)
PAOD	0.596		
Current smoker	0.906		
EuroSCORE II	<0.001	0.107	1.03 (0.99–1.06)
NSTEMI	0.056	0.176	1.81 (0.77–4.29)
Left main disease	0.182	0.203	0.54 (0.21–1.39)
Number of anastomoses	0.387		

With the essential variable being ‘use of ticagrelor’, variables that showed *P* values less than 0.2 were included in multivariable analysis. HR, hazard ratio; NSTEMI, non-ST-elevation myocardial infarction; PAOD, peripheral artery occlusive disease. ^a^ *P* values less than 0.05.

**Table 5:** Standardized mean difference (SMD) between the groups before and after propensity score matching.

	ASA + CPD (*n* = 100)	ASA + TCG (*n* = 169)	SMD (Before Matching)	ASA + CPD (*n* = 76)	ASA + TCG (*n* = 76)	SMD (After Matching)
Age (years)	64 ± 10	64 ± 10	2%	64 ± 10	64 ± 9	3%
Sex (male)	80 (80%)	121 (72%)	19%	59 (78%)	56 (74%)	9%
BSA (m^2^)	1.7 ± 0.2	1.7 ± 0.2	9%	1.7 ± 0.2	1.7 ± 0.2	11%
Diabetes	52 (52%)	86 (51%)	2%	40 (53%)	36 (47%)	11%
Hypertension ^a^	66 (66%)	89 (53%)	27%	45 (59%)	44 (58%)	3%
Creatinine clearance (mL/min) ^a^	66 ± 33	76 ± 30	32%	69 ± 34	73 ± 30	14%
Atrial fibrillation ^a^	11 (11%)	7 (4%)	34%	6 (8%)	6 (8%)	0%
Old MI	13 (13%)	13 (8%)	20%	6 (8%)	9 (12%)	15%
Recent PCI	5 (5%)	13 (8%)	10%	3 (4%)	4 (5%)	5%
Current smoker	31 (31%)	47 (28%)	7%	26 (34%)	23 (30%)	9%
EuroSCORE II (%) ^a^	9.5 ± 8.7	7.7 ± 7.1	25%	8.1 ± 6.3	7.8 ± 7.4	4%
NSTEMI ^a^	49 (49%)	114 (68%)	39%	42 (55%)	39 (51%)	8%
Left main disease	33 (33%)	46 (27%)	13%	22 (29%)	23 (30%)	3%
Number of anastomoses	3.6 ± 0.9	3.7 ± 1.0	15%	3.5 ± 0.9	3.5 ± 1.1	1%
Urgent operation	4 (4%)	6 (3.6%)	2%	3 (4%)	3 (4%)	0%

^a^ Variables that exhibited a large imbalance (>25%) before matching. ASA, acetylsalicylic acid; BSA, body surface area; CPD, clopidogrel; MI, myocardial infarction; PCI, percutaneous coronary intervention; SMD, standardized mean difference; TCG, ticagrelor.
